# New Year

**Published:** 1851-01

**Authors:** 


*New Year.*—With this number a new year opens. To our many personal friends embraced among the patrons of our journal, and to our uumerous readers who are personally unknown to us, we tender, most corpially and sincerely, the salutation, of the season, 'a happy new year!'

